# Testosterone Levels Are Negatively Associated with Childlessness in Males, but Positively Related to Offspring Count in Fathers

**DOI:** 10.1371/journal.pone.0060018

**Published:** 2013-04-03

**Authors:** Thomas V. Pollet, Kelly D. Cobey, Leander van der Meij

**Affiliations:** 1 Department of Social and Organizational Psychology, VU University Amsterdam, Amsterdam, The Netherlands; 2 Department of Social Psychology, University of Groningen, Groningen, The Netherlands; Max-Delbrück Center for Molecular Medicine (MDC), Germany

## Abstract

Variation in testosterone (T) is thought to affect the allocation of effort between reproductive and parenting strategies. Here, using a large sample of elderly American men (n = 754) and women (n = 669) we examined the relationship between T and self-reported parenthood, as well as the relationship between T and number of reported children. Results supported previous findings from the literature, showing that fathers had lower T levels than men who report no children. Furthermore, we found that among fathers T levels were positively associated with the number of children a man reports close to the end of his lifespan. Results were maintained when controlling for a number of relevant factors such as time of T sampling, participant age, educational attainment, BMI, marital status and reported number of sex partners. In contrast, T was not associated with either motherhood or the number of children women had, suggesting that, at least in this sample, T does not influence the allocation of effort between reproductive and parenting strategies among women. Findings from this study contribute to the growing body of literature suggesting that, among men, pair bonding and paternal care are associated with lower T levels, while searching and acquiring sex partners is associated with higher T levels.

## Introduction

Humans are among a relatively restricted group of mammals, 5% or less, that display male paternal care [1], [2]. It is not uncommon for fathers to provide direct care to dependent offspring well into the second decade of their life, often even caring for multiple overlapping offspring at the same time (e.g., [3], [4]). As a consequence of paternal investment human males must regulate the time and energy they allocate between mating and parental effort [2], [3].

Wingfield, Hegner, Duffy, and Ball’s ‘challenge hypothesis’, based on research from bird species, suggests that testosterone (T) helps to regulate the trade-off between mating and parenting [5]. Specifically, it explains the function of varied T levels in seasonally breeding birds, wherein T levels rise in the mating season and then subsequently drop during the period of brooding and parental care. The challenge hypothesis, reviewed in [6], has since been adapted to also account for changes in mating and parenting effort in humans (review in [7], example: [8]). Of course, in humans, breeding does not follow a seasonal pattern, instead human males experience high chronic T levels and can pursue mating opportunities throughout their lifespan [9]. Nonetheless, research on the life history of human males appears to support the idea that T is intricately involved in regulating mating and pair bonding/parenting behavior (for review: [10]). Low male T levels have been shown to promote features relevant to parenting or investment [7]. For example, married men are known to have lower T levels than samples of matched men who are unmarried (e.g., [11]–[14]). Likewise, men who are in committed relationships have been shown to have lower levels of T than uncommitted single men (e.g., [14]–[16]). A complementary line of research has shown that the transition to fatherhood is also associated with a decrease in T levels (e.g., [13], [17]–[20]). The role of low T in facilitating parenting seems to be robust: in non-Western populations fathers have also been found to have lower T levels than non-fathers [17], [19]–[21]. The adjustment of T levels upon the transition to fatherhood has been proposed to facilitate better fathering through priming men to provide care [22]. Yet, T levels have been found to be negatively related to paternal investment in three studies from non-Western cultures [17], [21], [23]. The notion of low levels of T being associated with nurturing responses is, however, consistent with recent laboratory based work by van Anders and colleagues [24] who showed that providing a nurturing response to a crying doll decreases male T levels. However, it should be noted that this sample consisted mostly of non-fathers. In line with van Anders et al. findings [24], men who express a greater need to comfort a crying baby experience an even larger decrease in their T levels than men who do not express such a need [22]. However, a different study showed an increase in T levels among fathers in response to crying by infants [25]. Moreover, to date, there is no conclusive evidence that T levels in males are negatively related to interaction with children. One study using data on Jamaican fathers [26] and one study on Filipino fathers [27] did not find any significant relationship between T levels and interaction with children. A study by Storey and colleagues found a negative relationship between amount of time spent interacting with toddlers and percent change in T levels in a sample of Canadian fathers [28].

In contrast to findings suggesting that low T levels are related to nurturing behaviors, high T levels are known to promote mating behaviors. For example, T levels are known to increase when men view sexually explicit videos [29] and after they have had sex [30]. Likewise, male T levels increase after a brief non-physical social interactions with women [8], [31]. Men with elevated T levels also show more affiliative behaviors towards women [32]. Finally, high T levels are positively associated with the number of sex partners a man reports [33]–[35], and, more broadly, have been argued to be positively related to male reproductive effort in humans [9].

There is also evidence that T levels regulate the tradeoff between mating and parental effort in women. For example, it has been shown that single women have higher levels of T than partnered women [36], married women have lower T levels than single women [37], and mothers have lower T levels than non-mothers [37], [38]. However, the evidence linking T levels to mating and parental effort is far scarcer for females than it is for males, especially when compared across species.

Here, we present data that allowed us to explore the relationship between being a parent, number of children and T levels in a sample of elderly men and women. To our knowledge, no study in humans has yet examined the relationship between T levels and breeding success in men and women with at least one child in a large sample. We predicted, in line with the aforementioned literature, that fatherhood and motherhood would be negatively associated with T levels. In addition, we predicted that, among fathers, breeding success would be higher among men with relatively higher levels of T as these men may be more inclined to focus on mating effort. Among mothers we also explored this latter relationship, but we did not expect a higher breeding success for women with high T levels, as it seems unlikely that women would increase their number of children via mating effort in the same way men do.

## Methods

### 1. Ethics Statement

This paper makes use of secondary data analysis of a previously collected data source, which is available to researchers via a data archive (ICPSR, www.icpsr.org). Ethical review was waived for the current research project by the Ethics Review Board (VCWE, Faculty of Psychology and Pedagogics, VU University Amsterdam). The ethics approval of the original data collection can be found in the NSHAP codebook (see: http://www.norc.org/Research/Projects/Pages/national-social-life-health-and-aging-project.aspx).

### 2. Dataset

Our predictions were tested using an archival dataset of older Americans aged 57–85 from the *National Social Life, Health, and Aging Project (NSHAP)* (N = 3,005; [39]). Relative to the American population the dataset oversampled men, African-Americans, Latinos, and the eldest age groups. Trained interviewers from the National Opinion Research Centre (NORC) conducted the face-to-face interviews between July 2005 and March 2006. Participant response rate was 75.5% (for the full description of the data collection see [39] or [40]). At the time of the interview participants also provided several in-home biomeasures (e.g. Testosterone, Cotinine) and were given a leave-behind questionnaire. We limited the sample to White participants (74% of the male working sample, *n* = 754*;* 70% of the female working sample *n* = 669), as exploring the relationship in other ethnicities left us with too few cases (Males: Hispanics: *n = *111; African Americans: *n = *129; Other *n* = 29; Females: Hispanics: *n = *108; African Americans: *n = *157; Other *n* = 18). While these numbers are much higher than some published samples for T and paternal behavior (e.g., [22]), given the high level of noise in these data and the small effects we expect at the end of participants’ reproductive lifespan, samples of around 150 are likely not sufficient. In addition, there are suggestions that ethnicities differ both in their T levels (e.g., [41]) as well as in their reproductive behavior (e.g., [42]–[44]). In ESM S1 we present the results for the full sample with all ethnicities. These results are quite similar to those reported below. However, given that the reported effects are predominantly driven by White participants (>70% of working sample) and the reasons outlined above, we have chosen to report the results for White participants rather than those of the pooled sample.

### 3. Testosterone

Ninety percent of the overall sample complied in providing saliva samples (N = 3,005). Participants who failed to have a saliva samples taken successfully were excluded (4.5% of overall sample failed to provide a sample or provided a sample which was not valid due to equipment failure (N = 135)). Saliva samples were transported from interviews using cold packs and dry ice and were shipped to Salimetrics for analysis in duplicate (75.3% of the saliva samples provided actual T values, see [39] for further details). The procedure and assay are described in [35] and [45]. We excluded outliers of two standard deviations above the mean (>166.75 pg/ml for males; >93.58 pg/ml for females). As the key independent variable we used the mean scores of the T values from both hormone assays, which we subsequently logarithmically transformed as values did not follow a normal distribution.

### 4. Childlessness and Number of Living Children

Number of children and childlessness were used as the dependent variable. This was captured by the items: *“How many living daughters do you have”* and *“How many living sons do you have”* ([39]: p. 32-ff.). When participants asked if they should include or exclude step-sons/daughters they were told that they could include them. It is thus possible that responses to these items include non-biological children. However, we believe that the error as a result from asking about and reporting non-biological children can be considered as random noise, which is unrelated to T levels. Therefore, it seems unlikely that this noise would influence the statistical conclusions of our results.

### 5. Control Variables

While some findings suggest that in older men the circadian rhythm in T is blunted or even lost (see [46]), we included the time of sampling as a control variable (coded as am/pm). Further, since male T levels are known to decrease with age [47], participant age was also used as a control variable. Educational attainment (four categories) and Body Mass Index (BMI; measured by a trained interviewer) were included as these may be factors which influence circulating T and perhaps its relation to childlessness or offspring count. Similarly, since previous research has shown that men with higher T levels are more likely to divorce [12] and that T influences the likelihood of remarriage [35] we controlled for marital status. Furthermore, it is possible that any relationship between childlessness and T is an artifact of these males being in a committed relationship. Thus, marital status is a necessary control variable to distinguish whether or not the effect is driven by differences in T or differences in marital status. We also explored if the number of opposite sex partners a participant reported influenced our main findings, because males with higher T have been shown to report more female sex partners (e.g., [33]–[35]). These findings are described in the ESM S2. The descriptive statistics of the aforementioned control variables can be found in ESM S3.

### 6. Statistical Analyses

We used Generalized linear models with Maximum Likelihood estimation to analyze the data. We use negative binomial regression to analyze childlessness (GZLM; with NBR, [48]). This method was preferred over logistic regression as it deals with overdispersion better. For individuals with at least one child, offspring count was analyzed via adjusted Poisson models, given that we are dealing with skewed count data. The number of children can be conceived as count data following a Poisson distribution (number of events in an unknown sequence). Indeed a Poisson model proved a much better fit to the data than a linear model (all models ΔAIC>50 (Poisson vs. linear), although the conclusions drawn from our results were the same for both models. The standard errors for the models we present were adjusted for model deviance, and thus take into account the under- or overdispersion in any given model [48], [49].

We reported model fit (AIC, BIC) and parameter statistics for our models. Our modeling strategy was as follows: (1) we first examined a baseline model with no control variables; (2) we then sequentially added control variables, and only maintained these variables when they improved model fit (Akaike Information Criterion (AIC); smaller-is-better; [50]). As a rule of thumb, two units indicates an indistinguishable difference between models, whereas ten units indicates a large difference between models and more support for model A over model B [51]. This procedure is an information theoretic approach which leads to the best fitting model from our given set of variables [52], [53]. Additionally, we reported also the Bayesian information criterion for each model (BIC, [54]) which tends to be more conservative. We presented the results separately for males and females, as the distributions for T and offspring count are expected to differ significantly between the sexes. All statistical tests were performed with SPSS version 16.0. Values are mean ± standard error means (SE) unless otherwise specified. For illustrative purposes, we used raw T scores or *Z* scores, rather than log (T) scores within the (online) figures.

## Results

### 1. Males: Childlessness

**Figure 1:**
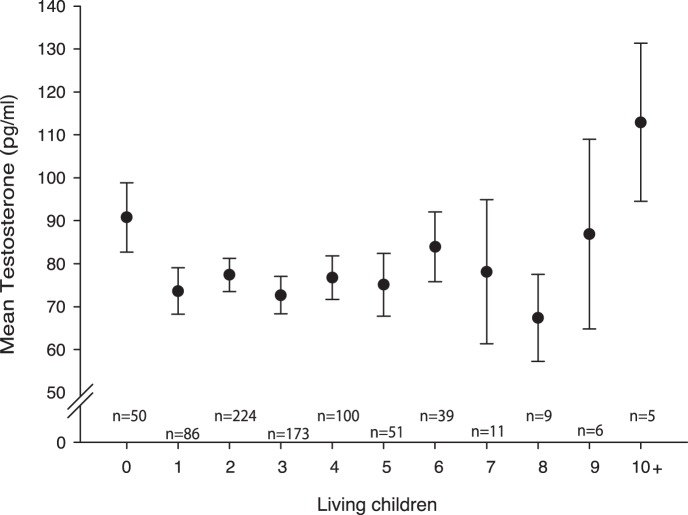
Number of living children and mean testosterone level for Caucasian men (n = 754). Error bars represent 95% Confidence Intervals (10+ is only used for graphical representation analyses used the full range).

Testosterone levels were associated with male childlessness (see Fig. 1 and Table 1). Men who reported no living children had higher T levels (90.79+/−4.11 pg/ml) than men with living children (76.46+/−1.04 pg/ml), see also Fig. 1. The Negative Binomial Regression Model showed indeed that log (T) was positively associated with childlessness. This effect remained after controlling for marital status (see model 2 in Table 1). However, the odds ratio (exp(B)) for log(T) dropped from 21.08 to 8.48 after inclusion of Marital Status, suggesting that the relationship between log(T) and childlessness is mediated by not being married. Model 2, with marital status and log(T) as predictors, could not be improved by adding the other control variables (ΔAIC<1).

**Table 1:** Parameter estimates (B), standard errors, Exp(B), and concomitant test statistics for Negative Binomial Regression models with childlessness as dependent variable.

Childlessness in Men			B	SE	Exp(B)	p
**Model 1**	log(testosterone)	(pg/ml)	3.048	0.5486	21.08	<.00001
**Model 2**	log(testosterone)	(pg/ml)	2.137	0.4956	8.475	<.00001
(ΔAIC = 53.40; ΔBIC = 34.90)	Marital Status	Married	−3.300	0.1970	0.037	<.00001
		With partner	−1.351	0.339	0.259	<.00001
		Divorced/separated	−2.328	0.2913	0.097	<.00001
		Widowed	−1.901	0.2298	0.149	<.00001
		Never Married	–	–		

For Marital status, ‘Never Married’ was set as reference category.

### 2. Males: Offspring Count among Men with Living Children

When examining only men with at least one child, male T levels were significantly and positively associated with the number of living children (see Figure 1 and Table 2). At baseline, the model predicts that an increase of one standard deviation from the mean in raw T levels amounts to an increase of.12 children (see ESM S4). The significant association between T and offspring count remained after controlling for age and educational attainment (see model 2 and 3 in Table 2). Older and lower educated men tended to have more children than younger and higher educated men respectively. Model 2 and Model 3 were virtually indistinguishable in terms of model fit (ΔAIC<2). Using a Bayesian approach, one would select model 2 over model 3 (ΔBIC>11). Model 3 could not be improved by adding other control variables, such as marital status (ΔAIC<1). As described in the endnote controlling for the number of reported opposite sex partners or remarriage does not alter this finding.

**Table 2:** Parameter estimates (B), standard errors, Exp(B), and concomitant test statistics for overdispersed Poisson Models with offspring count as dependent variable.

Offspring count of men with at least one child			B	SE	Exp(B)	p
**Model 1**	log(testosterone)	(pg/ml)	0.243	0.113	1.275	0.032
**Model 2**	log(testosterone)	(pg/ml)	0.294	0.113	1.341	0.009
(ΔAIC = 13.75; ΔBIC = 9.2)	age	(years)	0.011	0.003	1.011	<.0001
**Model 3**	log(testosterone)	(pg/l)	0.293	0.112	1.341	0.009
(ΔAIC = 1.74; ΔBIC = −11.81)	age	(years)	0.010	0.003	1.010	0.0002
	Education	<High School	0.175	0.066	1.191	0.008
		High School	0.125	0.052	1.133	0.015
		Voc./college/…	0.076	0.051	1.079	0.14
		Bachelors or more	–	–	–	–

For educational attainment ‘Bachelors or more’ was set as reference category.

### 3. Females: Childlessness and Offspring Count

For women there was no association between log (T) and childlessness (Negative Binomial Regression Model; B = .572+/−.44; *χ^2^* test = 1.694; *p* = .193). Similarly, there was no association between log (T) and offspring count among women who reported living children (Poisson Model; B = 0.071+/−109; *χ^2^* test = 0.424; *p* = .515).

## Discussion

Three main findings emerge from this research. Firstly, we replicated the finding that fathers have lower T levels than non-fathers (e.g., [13], [17]–[19], [22]). However, to our knowledge, this is the first time that this has been demonstrated in a large sample of elderly men. The decrease in T levels upon the transition to fatherhood is thought to facilitate the allocation of energy to activities related to parenting. Yet, there are several studies indicating that T levels can be *negatively* related to paternal investment in males [17], [21], [23]. In contrast, the notion that T is positively related to fathering is supported, for example, by the finding that men who experience a greater need to comfort a crying baby experience a larger decrease in their T levels [22]. It is also consistent with findings that providing a nurturing response to crying baby actually decreases male T levels [24], though the majority of this sample consisted of non-fathers. However, it is still unclear whether T levels and interacting with offspring are consistently related in males (see [26]–[28]). Our results add to the current literature by showing that in old age there still is a marked significant difference between (self-reported) fathers and non-fathers in T levels, though from this cross-sectional study it is unclear what the mechanism driving this decrease among fathers is. One potential mechanism could be close interaction with young grandchildren, rather than fatherhood status per se. However, the finding that (putative) fatherhood is associated with low T levels in old age, seems at odds with recent findings by Gettler and colleagues who showed that while T levels decreased when Filipino men became fathers, T levels returned to similar levels before fatherhood as their children aged [17]. These findings suggest that longer-term effects of fatherhood on T levels are perhaps not universal and that they may differ between Western and non-Western societies.

Secondly, we showed that among elderly men who reported being fathers, those with higher T levels reported more (living) children than those with lower T levels. To our knowledge, our study is the first to demonstrate in a large sample of putative fathers that T levels are related to the reported number of children towards the end of man’s reproductive lifespan. While in theory, men in our sample may still have more offspring, they are likely to be close to the end of their reproductive lifespan as they were on average 69 years old. That fathers with higher T levels would have more children is consistent with the predictions of the Challenge Hypothesis [7] and with T being involved in mating effort in humans [9]. According to both these perspectives, in contexts where mating effort is required, e.g. to acquire sex partners, T levels are (chronically) higher. Consequently, those fathers with higher T levels may engage more frequently in attracting (additional) partners for sex, thereby increasing their likelihood to father additional children. This may be accomplished either through increased promiscuity, extramarital affairs or new marriages. Indeed, in this same dataset we have previously shown that men with higher T levels report more sex partners [35]. Similarly, previous research has shown that men with higher T levels are more likely to get divorced [12]. Taken together, these behaviors may result in the fathering of more children during their lifespan than fathers who did not engage in the acquisition of more sex partners. However, our results did not show that fathers with high T levels had more children because they remarried or because they had more sex partners. It may be that this effect was obscured because of social desirability in responses, which could especially cause men with a family (i.e., fathers) to underreport extramarital affairs.

Apart from the acquisition of more sex partners by men with high T levels, additional mechanisms are plausible. It could be that those fathers with high T levels were more fertile than those with lower T levels. In line with this reasoning, it is well established that T is crucial for male reproductive effort, fertility and the maintenance of spermatogenesis ([9], [10], [55] for mechanisms see [56], [57]) and, not surprising, low T levels are associated with male infertility [58]. However, when considering fertile men, a recent study found no relationship between semen quality and T levels [59]. This latter finding suggests that increased fertility is not a likely explanation for our finding. It could be that, although a certain level of T is mandatory for fertility, higher T levels do not lead to increased fertility among men who are already fertile. Furthermore, rather than a direct effect of T on fertility, there could also be a number of indirect effects of T on the number of children a man has. For example, lower T levels are positively associated with obesity, stress and exposure to industrial pollutants (reviewed in [60]). It could thus be that those men with high T levels have more children since they are more physically motivated and capable, and thus experience more opportunities for mating than men with lower T levels. However, this relationship does not seem that likely since we controlled for BMI, which is an important health indicator.

Finally, we found that T levels were not associated with either motherhood or number of living children in our female sample. This finding is in contrast to the results of Kuzawa and colleagues [38]. However, it is possible that among women there are no measurable associations between T and childlessness or offspring count at an older age, while these do exist at other periods throughout a female’s life span, as the findings by Kuzawa and colleagues on women in their reproductive window suggest. In this respect, it is important to note that androgen levels of postmenopausal women are substantially different from those of women of reproductive age (e.g, [61]). Moreover, the population and context of the Philippines, is very different from that of the United States, which likely makes the populations hard to compare (see [62], [63]). Therefore these findings are not necessarily at odds with those of Kuzawa and colleagues [38].

There are several notable limitations to this study. Firstly, our conclusions are based on self-reports. It could be that men with higher T levels are more prone to boast their number of children, or alternatively, be more likely to include stepchildren as their own children. However, this seems unlikely since the effect of T on the number of self-reported children was not mediated via remarriage. Secondly, this study could not disentangle the mechanism through which fathers with higher T levels had more children: it could be that they had more sex partners (although they did not report them), that they were more fertile, that they had sex with their partner more frequently, or that they preferred sex partner(s) who wanted relatively more children. Similarly, men with high T levels might have actually paired with more fertile women. Thirdly, the fathers in our study were not yet at the end of their theoretical reproductive life span and could therefore conceive additional children. The survey items also specifically asked participants to report on the number of *living* children. One implication of our this finding is that fathers with high T could invest less time towards parenting effort, which could potentially reduce the quality, health or even survival of their offspring. If it is true that survivorship of children was affected by high T fathers, findings reported herein may be somewhat underestimated. Finally, our interpretation of the relationship between number of offspring and T levels assumes that individual differences in T levels demonstrate some stability over time. Indeed, there is some evidence that this so, since it has been shown in a longitudinal study covering 30 years that male free T is at least moderately correlated (*r* = 0.5, [64]). The results from this study contribute to the growing literature that shows that pair bonding and paternal care are associated with low T levels, while searching for and acquiring sex partners is associated with high T levels. An interesting avenue to pursue in future research could be to look more closely at how age of children or contact with children might interact with the effects reported herein. Future research on T and reproductive success should take into account that while fatherhood may decrease T levels in both the short and long term, once a father, higher T levels are positively associated with number of children.

## Supporting Information

ESM S1 Additional analyses on the full sample.
(DOCX)

ESM S2 Additional analyses on sample of [35].
(DOCX)

ESM S3 Descriptive statistics for the male working sample (n = 754) and the female working sample (n = 669).
(DOC)

ESM S4 Predicted number of children in Poisson Model by Z scores of (raw) testosterone for childed men (n = 704).
(TIF)
