# Correction: Testosterone Levels Are Negatively Associated with Childlessness in Males, but Positively Related to Offspring Count in Fathers

**DOI:** 10.1371/annotation/bccccb7e-48a7-4594-b3e6-ce8c9d2489a2

**Published:** 2013-06-27

**Authors:** Thomas V. Pollet, Kelly D. Cobey, Leander van der Meij

There was an error in the article title. The correct title is: "Testosterone Levels Are Negatively Associated with Fatherhood in Males, but Positively Related to Offspring Count in Fathers." 

The correct citation is: Pollet TV, Cobey KD, van der Meij L (2013) Testosterone Levels Are Negatively Associated with Fatherhood in Males, but Positively Related to Offspring Count in Fathers. PLoS ONE 8(4): e60018. doi:10.1371/journal.pone.0060018. 

