# Supplementary material for: Testosterone Levels Are Negatively Associated with Childlessness in Males, but Positively Related to Offspring Count in Fathers
Source: PLoS One. 2013 Apr 3;8(4):e60018. doi: 10.1371/journal.pone.0060018 (PMC3616053; doi:10.1371/journal.pone.0060018)
Supplement: ESM S1 — Additional analyses on the full sample. (DOCX) [file pone.0060018.s001.docx]

**S-ESM 1. Additional analyses on full sample.**

**1) Childlessness**

- 1. **Males**

| **Childlessness in Men** |  |  | **B** | **SE** | **Exp(B)** | **p** |
| --- | --- | --- | --- | --- | --- | --- |
| **Model 1** | log(testosterone) | (pg/ml) | 1.227 | 0.4173 | 3.412 | <.00001 |
| **Model 2** | log(testosterone) | (pg/ml) | 1.382 | 0.4125 | 3.983 | .00081 |
| (ΔAIC= 133.72; ΔBIC= 115.69) | Marital Status | Married | -3.181 | 0.1716 | 0.042 | <.00001 |
|  |  | With partner | -1.654 | 0.3273 | 0.191 | <.00001 |
|  |  | Divorced/separated | -2.028 | 0.216 | 0.132 | <.00001 |
|  |  | Widowed | -1.945 | 0.2046 | 0.143 | <.00001 |
|  |  | Never Married | - | - |  |  |
| **Model 3** | log(testosterone) | (pg/ml) | 1.551 | 0.4195 | 4.717 | .00022 |
| (ΔAIC= 0.27; ΔBIC= -5.68) | Marital Status | Married | -3.141 | 0.1715 | 0.043 | <.00001 |
|  |  | With partner | -1.605 | 0.3274 | 0.201 | <.00001 |
|  |  | Divorced/separated | -2 | 0.2159 | 0.135 | <.00001 |
|  |  | Widowed | -2.045 | 0.2092 | 0.129 | <.00001 |
|  |  | Never Married | - | - |  |  |
|  | Age | (years) | 0.021 | 0.0089 | 1.021 | .01872 |

Results for Negative Binomial regression models for childlessness in men. See full text for description. Results for full working sample including males of all ethnicities (N=1,043). This model cannot be improved in terms of AIC/BIC by adding further variables.

**1.2 Females**

There is no effect of log(T) on childlessness in full working sample including females of all ethnicities (B= -.216; SE=0.468; Wald=.212 ; p= .645; N=1,006).

**2) Offspring count**

**2.1 Males**

| **Offspring count of men with at least one child** | | | **B** | **SE** | **Exp(B)** | **p** |
| --- | --- | --- | --- | --- | --- | --- |
| **Model 1** | log(testosterone) | (pg/ml) | 0.253 | 0.1 | 1.288 | 0.012 |
| **Model 2** | log(testosterone) | (pg/ml) | 0.169 | 0.097 | 1.184 | 0.081 |
| (ΔAIC= 57.04; ΔBIC=52.16) | Ethnicity | (Non-White --> White) | -0.292 | 0.037 | 0.747 | <.0001 |
| **Model 3** | log(testosterone) | (pg/l) | 0.154 | 0.095 | 1.166 | 0.105 |
| (ΔAIC= 34.88; ΔBIC=20.23) | Ethnicity | (Non-White --> White) | -0.195 | 0.04 | 0.823 | <.0001 |
|  | Education | <High School | 0.312 | 0.052 | 1.366 | <.0001 |
|  |  | High School | 0.125 | 0.049 | 1.134 | 0.011 |
|  |  | Voc./college/… | 0.042 | 0.049 | 1.043 | 0.392 |
|  |  | Bachelors or more | - | - | - | - |
| **Model 4** | log(testosterone) | (pg/l) | 0.187 | 0.095 | 1.21 | 0.049 |
| (ΔAIC= 11.42; ΔBIC=6.54) | Ethnicity | (Non-White --> White) | -0.207 | 0.04 | 0.813 | <.0001 |
|  | Education | <High School | 0.288 | 0.052 | 1.334 | <.0001 |
|  |  | High School | 0.108 | 0.049 | 1.114 | 0.028 |
|  |  | Voc./college/… | 0.042 | 0.049 | 1.043 | 0.387 |
|  |  | Bachelors or more | - | - | - | - |
|  | Age | (years) | 0.008 | 0.002 | 1.01 | <.0001 |

Results for Overdispersed Poisson regression models for offspring count in men with at least one child. See full text for description. Results for full working sample including males of all ethnicities (N=974). This model cannot be improved in terms of AIC/BIC by adding further variables.

**2.2 Females**

There is no effect of log(T) on number of children in full working sample including females of all ethnicities with at least one child (B= -.026; SE= 0.076; Wald=.121; p= .728; N=929).
