# Supplementary material for: Testosterone Levels Are Negatively Associated with Childlessness in Males, but Positively Related to Offspring Count in Fathers
Source: PLoS One. 2013 Apr 3;8(4):e60018. doi: 10.1371/journal.pone.0060018 (PMC3616053; doi:10.1371/journal.pone.0060018)
Supplement: ESM S2 — Additional analyses on sample of [35]. (DOCX) [file pone.0060018.s002.docx]

**Additional analyses on sample of [35]**

We have previously shown that T relates to the number of opposite sex partners reported in men [35]. The results are the same when this more restricted sample is used and when we control for opposite sex partners ceiled to 50 (Effect of log(T) on male childlessness: B= 3.455+/- 0.707; χ²= 23.89; p<.0001; Effect of log(T) on offspring count for childed men: B= 0.365+/-0.139; χ²=6.94; p=.008). Similarly, controlling for remarriage does not alter these findings (B= 4.35+/- 0.814; χ²= 23.89; p<.0001; Effect of log(T) on offspring count for childed men: B= 0.319+/-0.137; χ²=5.45; p=.02) Given that the samples differed by 10% and that number of opposite sex partners did not affect the results, we do not report this as a separate control variable but chose to report on the more complete sample. Nonetheless using the restricted sample and/or controlling for opposite sex partners or remarriage does not alter our conclusions. It is important to note that this sample excluded men who had sex with men [35], our results with the more complete sample are thus unlikely to be driven by a subsample of men with a homosexual preference.
