# Supplementary material for: Testosterone Levels Are Negatively Associated with Childlessness in Males, but Positively Related to Offspring Count in Fathers
Source: PLoS One. 2013 Apr 3;8(4):e60018. doi: 10.1371/journal.pone.0060018 (PMC3616053; doi:10.1371/journal.pone.0060018)
Supplement: ESM S3 — Descriptive statistics for the male working sample (n = 754) and the female working sample (n = 669). (DOC) [file pone.0060018.s003.doc]

| **Variables (Males)** | **Categories/Units** | **Frequencies / Means** | **% or SD** |
| --- | --- | --- | --- |
| Saliva collection time | Am | 186 | 24.7 |
|  | Pm | 568 | 75.3 |
| Education | less than high school | 86 | 11.4 |
|  | high school or equivalent | 200 | 26.5 |
|  | vocational qualification or some college | 219 | 29.0 |
|  | bachelors or more | 249 | 33.0 |
| Testosterone | (pg/ml) | 77.41 | 28.11 |
| Age | (years) | 69.03 | 7.80 |
| BMI | (kg/m²) | 29.05 | 5.29 |
| Marital Status | Married | 595 | 78.9 |
|  | Living with partner | 16 | 2.1 |
|  | Divorced/ Separated | 51 | 6.8 |
|  | Widowed | 73 | 9.7 |
|  | Never married | 19 | 2.5 |
| Offspring count | (number, if at least one child) | 3.12 | 1.74 |
| Childlessness | Childed | 704 | 93.4 |
|  | Childless | 50 | 6.6 |
| **Variables (Females)** | **Categories/Units** | **Frequencies / Means** | **% or SD** |
| Saliva collection time | Am | 166 | 24.8 |
|  | Pm | 503 | 75.2 |
| Education | less than high school | 87 | 13 |
|  | high school or equivalent | 215 | 32.1 |
|  | vocational qualification or some college | 222 | 33.2 |
|  | bachelors or more | 145 | 21.7 |
| Testosterone | (pg/ml) | 44.22 | 18.11 |
| Age | (years) | 69.9 | 8.88 |
| BMI | (kg/m²) | 28.38 | 6.05 |
| Marital Status | Married | 356 | 53.6 |
|  | Living with partner | 11 | 1.6 |
|  | Divorced/ Separated | 78 | 11.6 |
|  | Widowed | 203 | 30.3 |
|  | Never married | 21 | 3.1 |
| Offspring count | (number, if at least one child) | 2.99 | 1.73 |
| Childlessness | Childed | 615 | 91.9 |
|  | Childless | 54 | 8.1 |

S-ESM 2: descriptive statistics for the male working sample (n=754) and the female working sample (n= 669)
