# Supplementary figures and images for: Testosterone Levels Are Negatively Associated with Childlessness in Males, but Positively Related to Offspring Count in Fathers
Source: PLoS One. 2013 Apr 3;8(4):e60018. doi: 10.1371/journal.pone.0060018 (PMC3616053; doi:10.1371/journal.pone.0060018)

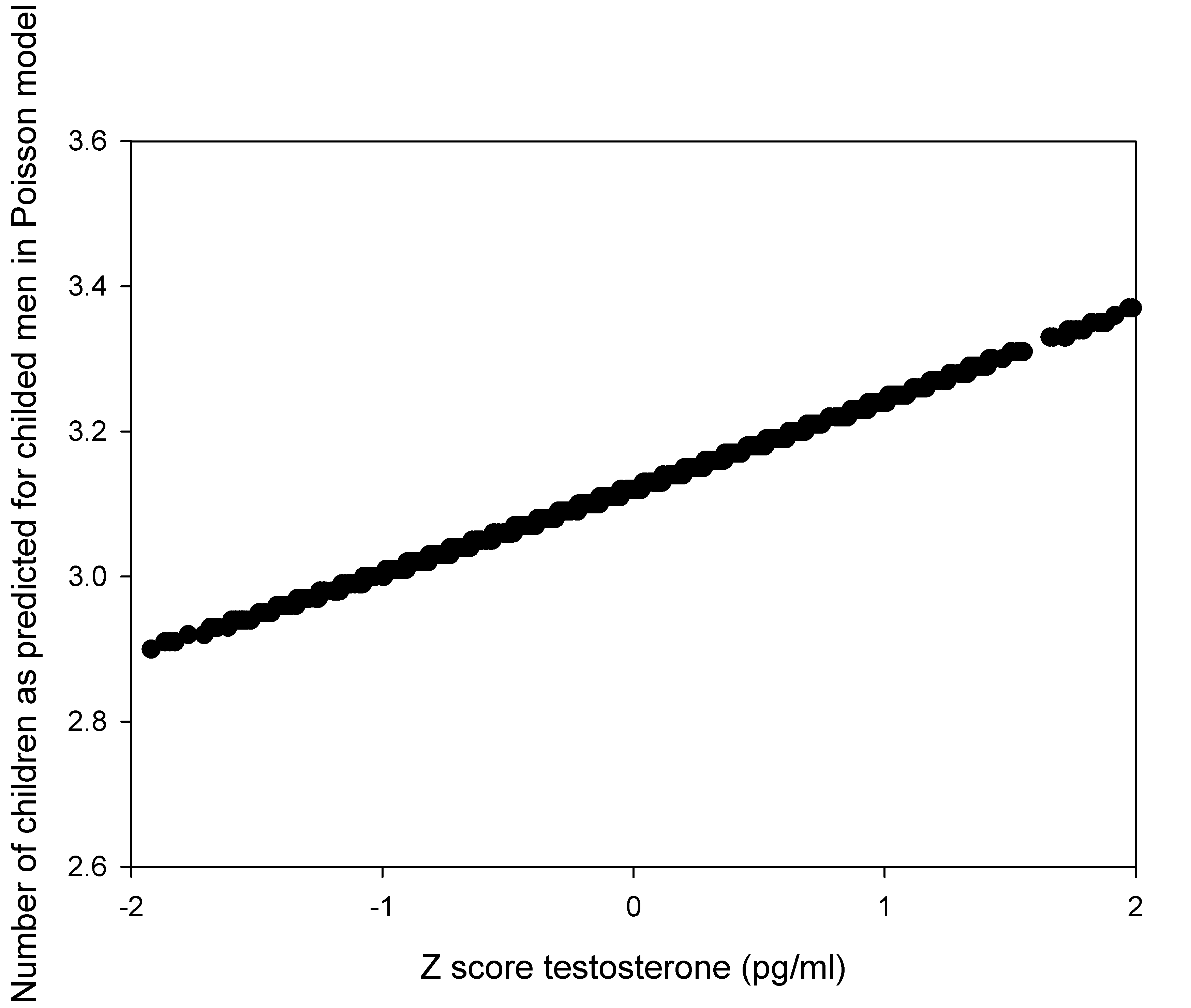

Supplement: ESM S4 — Predicted number of children in Poisson Model by Z scores of (raw) testosterone for childed men (n = 704). (TIF) [file pone.0060018.s004.tif]
